# Synthesis and Conformational Study of a Novel Macrocyclic Chiral(Salen) ligand and its Uranyl and Mn Complexes

**DOI:** 10.3390/molecules15031442

**Published:** 2010-03-09

**Authors:** Maria E. Amato, Francesco P. Ballistreri, Andrea Pappalardo, Gaetano A. Tomaselli, Rosa M. Toscano

**Affiliations:** Dipartimento di Scienze Chimiche, Università di Catania, Viale Andrea Doria 6, Catania 95125, Italy; E-Mails: eamato@unict.it (M.E.A.); fballistreri@unict.it (F.P.B.); andrea.pappalardo@unict.it (A.P.); rmtoscano@unict.it (R.M.T.)

**Keywords:** macrocyclic chiral ligand, salen, enantioselectivity, epoxidation

## Abstract

A novel chiral macrocyclic ligand incorporating a chiral salen moiety into a framework containing two biphenyl units was synthesized. Structural properties and conformational aspects of the free ligand and an UO_2_ complex were studied by using NMR spectroscopy in solution and MM calculations. The Mn(III) complex was tested as catalyst in enantioselective oxidation of prochiral unfunctionalized olefins to the corresponding optically active epoxides under very mild conditions.

## 1. Introduction

The successful design, synthesis and use of molecules capable of the selective recognition of other species is of great interest in catalysis, separations, enzyme functions and other fields involving molecular recognition [1]. Chiral salen-metal complexes are currently recognized versatile, practical and efficient catalysts for a large number of asymmetric reactions [2,3,4,5,6,7,8,9,10,11,12]. Moreover, in recent years, much attention has been devoted to the synthesis of new neutral ditopic salen receptors able to simultaneously bind chiral ammonium cations and their counteranions [13]. Generally, in addition to electrostatic interactions, hydrogen bonding and dispersive non bonding π-π interactions cooperate for binding affinity of intimate contact ion pair in organic solvents.

In this paper we describe the synthesis of a novel 21-membered macrocyclic ligand incorporating a chiral salen moiety into a framework containing two biphenyl units. The salen moiety, due to the presence of two stereogenic carbon atoms in the diimine bridge, generates a chiral pocket which can coordinate metal cations (*via* imine nitrogen and oxygen phenolic atoms), such as uranyl or Mn. In particular the uranyl cation can be employed as a Lewis acidic site able to bind ion pairs [13] whereas the Mn metal center can act as a catalytic site in the enantioselective epoxidation of olefins [14].

## 2. Results and Discussion

**Scheme 1:**
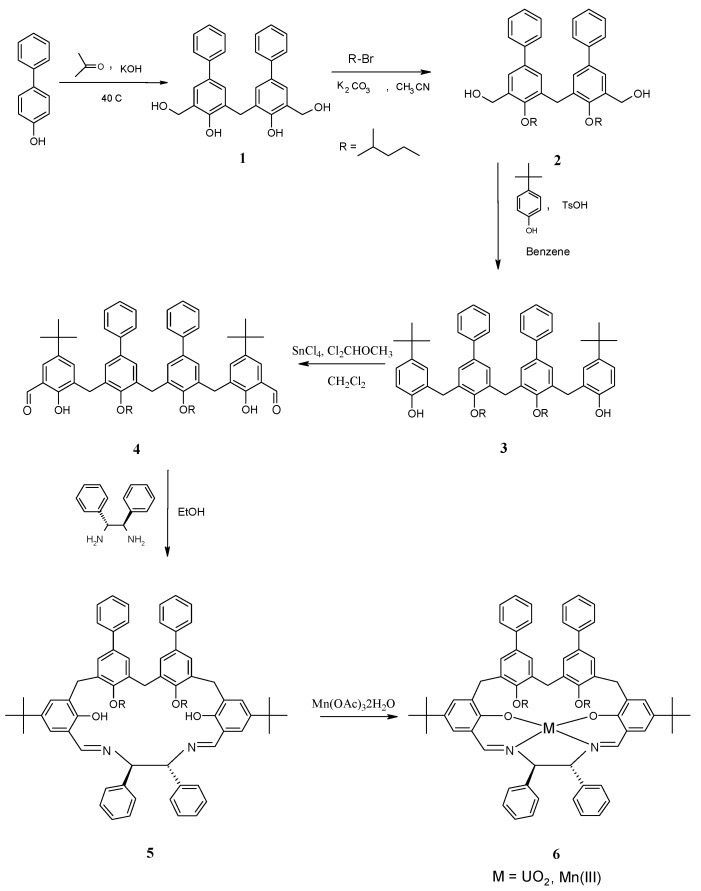
Synthesis of the macrocyclicsalen **5** and its metal complexes.

Synthesis of the macrocyclic diimine ligand **5** is shown in Scheme 1. The bis(hydroxymethyl) dimer **1** was prepared in 40% yield as reported elsewhere [15]. The selective alkylation of both phenolic hydroxyl groups with (CH_3_)_2_CH(CH_2_)_3_Br in refluxing dry acetonitrile in the presence of one equivalent of potassium carbonate as a base, afforded **2**, which was purified by column chromatography (67% yield).

Compound **2** was converted into **3** by acid-catalyzed condensation with a large excess of p-*t*-butyl-phenol in the presence of TsOH in benzene giving after column chromatography a white solid (76% yield). The subsequent formylation [14] of **3** afforded dialdehyde **4** (70% yield), which finally was cyclized with (1*R*,2*R*)-1,2-diphenylethylenediamine in refluxing ethanol under high diluting conditions (40% yield) to yield the macrocycle **5**.

The structural characterization of all new compounds **2**-**4** was achieved by ESI-MS measurements and 1D and 2D-NMR investigations. The ^1^H- and ^13^C­NMR spectra of compounds **2**, **3** and **4** in CDCl_3_ consist of relative simple patterns of resonances showing only one set of signals for each pair of identical groups. The formation of the macrocyclic ligand **5 **was fully supported by the ESI-MS measurements (m/z = 1,077 [MH]^+^). 

**Figure 1:**
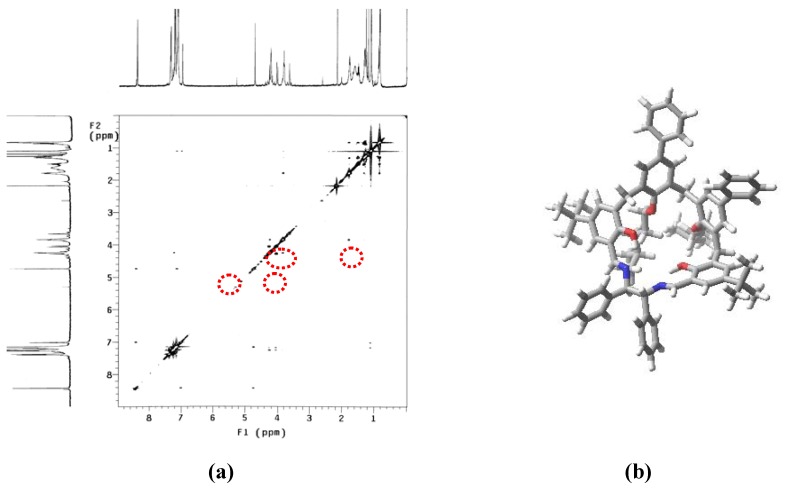
(**a**) ^1^H-NMR (500 MHz, CDCl_3_, 300K) T-ROESY map and (**b**) computed lowest-energy structure of macrocycle **5**.

The ^1^H-NMR spectrum of **5 **in CDCl_3_ exhibits two sharp singlet signals at 8.42 and 4.73 ppm assigned to the azomethine CH=N protons and the diimine bridge protons, respectively, while the broad singlet at 13.36 ppm arise from phenolic hydroxylic protons. The AB system centered at 4.24 ppm (Δδ = 0.05 ppm, *J* = 16.9 Hz) was assigned to the diasterotopic methylene protons located between the two *p*-phenyl-phenoxy groups. The AB system centered at 4.12 ppm (Δδ = 0.22 ppm, *J* = 15.8 Hz) integrating for four protons was easily attributed to the remaining two methylene groups. Aromatic protons resonate in the appropriate low field region and alkyl ether substituents gave one set of the expected multiplets (1–2 ppm). This simple spectrum, supported by the T-ROESY data (Figure 1a), indicates that the ligand assumes a C1 averaged symmetric cyclic structure in a non-interconvertible cone conformation ensured by the presence of sufficiently bulky 4-methylbutyl groups at the lower rim. In agreement with NMR spectroscopic data, MMFFs force field [16] calculation produced the computed lowest-energy structure of macrocycle **5 **(Figure 1b).

Ligand **5** was utilized to prepare both UO_2_(VI) and Mn(III) complexes **6-UO_2_** and **6-Mn** (Scheme 1). To a stirred solution of ligand **5** in EtOH, solid (AcO)_2_UO_2_•2H_2_O or Mn(AcO)_3_•2H_2_O respectively was added. The mixtures were allowed to stir overnight at room temperature and were monitored by TLC. Evaporation of the solvent gave a residue, which was dissolved in CH_2_Cl_2_, filtered and concentrated to produce the uranyl or Mn complex in a nearly quantitative yield. ESI-MS spectra confirmed the formation of the mono-metallic complexes.

**Figure 2:**
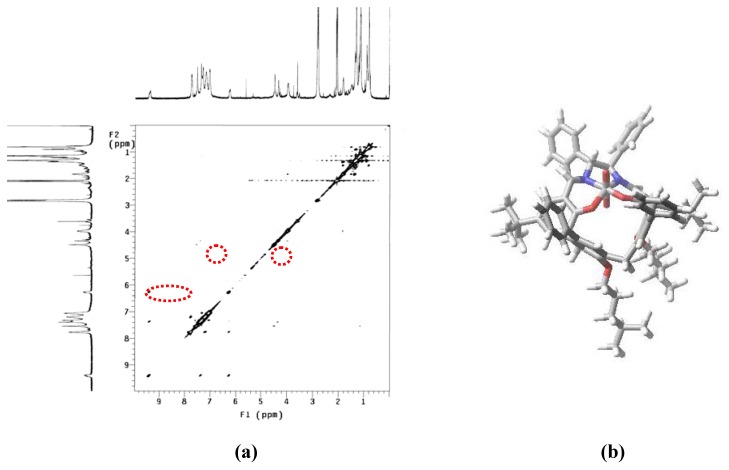
(**a**) ^1^H-NMR (500 MHz, (CD_3_)_2_CO, 300K) T-ROESY map and (**b**) modelled structure of complex **6-UO_2_**.

Structural information on the uranyl(VI) complex (**6-UO_2_** ), in solution, were obtained from 1D and 2D-NMR studies. The ^1^H-NMR spectrum in acetone-d_6_ of **6-UO_2_** complex showed an almost unvaried pattern of signals with respect to the free ligand, even considering the expected broadening and downfield shift of the resonances arising from the protons close to the coordination site. The ROE relationships measured by the phase-sensitive T-ROESY spectrum in acetone-d_6_ are shown in Figure 2a. The dipolar correlation contacts identified in the 2D map suggested that the symmetrical structure is maintained in solution upon metal complexation. The strong ROE correlations between aromatic protons signals and azomethine CH=N protons, diimine bridge protons and *t*-Bu groups allowed safe assignment of the partially overlapped signals of the aromatic protons system. Besides the characteristic bond-through ROE correlations in the aromatic moiety, no additional dipolar contacts were observed either between aromatics rings or between the aromatic protons and iso-hexyloxy-substituents. Interestingly, this finding ruled out any complex conformation where the diimine bridge phenyl rings were arranged in an almost *anti-periplanar* conformation. In agreement with MM calculated lowest energy structure, the complex adopts in solution a less hindered “disk-shaped” rather a “cup-like” shape (Figure 2b).

**Figure 3:**
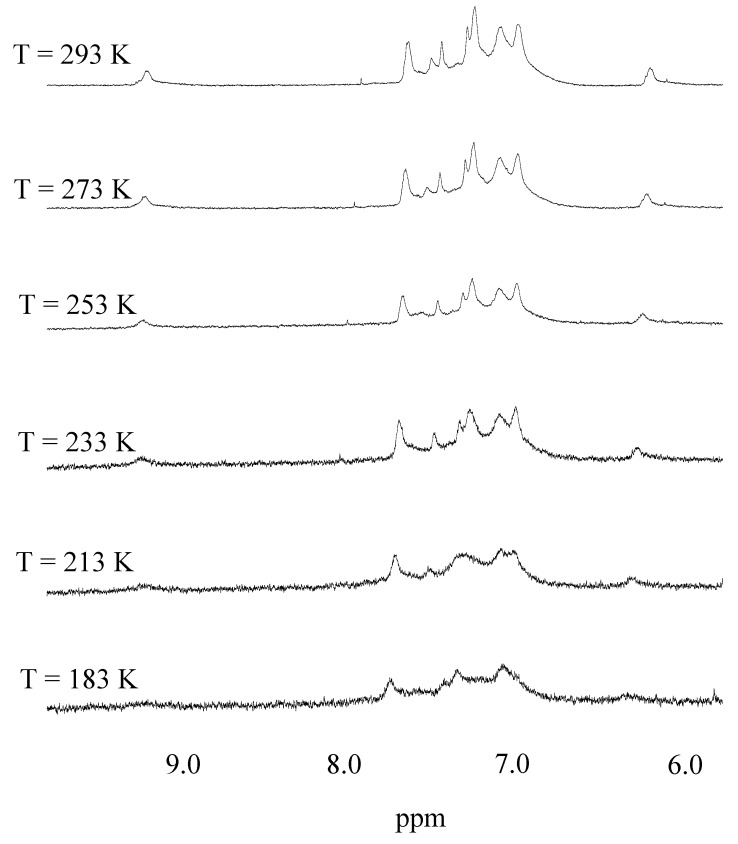
Variable temperature ^1^H NMR spectrum of **6-UO_2_** complex (500 MHz, (CD_3_)_2_CO) at relevant temperatures.

The broadening of the lines of the ^1^H spectrum might envisage the flipping motion of the salicylaldehyde framework already observed for uranyl salophen and salen complexes [13,17,18,19]. In order to enlighten on possible exchanging process occurring in solution, the proton signals of the uranyl complex were monitored carefully at lower temperature (Figure 3). As the temperature was lowered all the signals progressively broadened. At 193 K the signals of CH=N and diimine bridge protons were no longer visible and methylene protons located between the macrocycle aromatic rings coalesced into an unresolved envelope. Further cooling to 183 K did not produce additional detectable changes in the spectral profile neither new signals could be revealed. Only trivial modifications were observed in the range 183-213 K in the aromatic spectra, easily attributed to slower bi-phenyl rotation.

The **6-Mn** complex was characterised by ESI mass spectroscopy (m/z = 1129 [M]^+^). The catalytic ability of **6-Mn** was tested in the epoxidation reactions of some prochiral alkenes. The reactions were performed in CH_2_Cl_2_/H_2_O at 25° using NaClO as oxygen donor and 4-phenyl-pyridine *N*-oxide (4-PPNO) as coligand. Enantiomeric excess values for the formation of epoxides were determined by capillary GLC analysis employing chiral columns [14] for 1,2-dihydronaphthalene, and by ^1^H-NMR in the presence of Eu(+)(hfc)_3_ reagent shift for chromene derivatives epoxides.

The results are reported in Table 1. The reactions were allowed to proceed overnight (24 h) reaching 80%–90% completion (entries **2–4**) and quantitative yield in the corresponding epoxides (entries **1–5**).

**Table 1:** Enantioselective Epoxidation reactions of Alkenes with NaClO catalyzed by **6-Mn** in CH_2_Cl_2_/H_2_O at 25 °C. ^a^

Entry	Alkene	Conv. (%)	Yield (%)	ee (%)	Conf.^c^
**1**	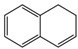	50^d^	90^b^	30^b^	1R,2S
**2**	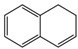	80	90^b^	50^b^	1R,2S
**3**	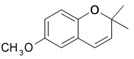	90^e^	100^e^	63^f^	3R,4R
**4**	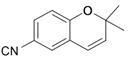	90^e^	100^e^	50^f^	3R,4R
**5**	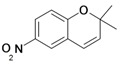	50^e^	100^e^	52^f^	3R,4R

^a^ In all experiments [Alkene] = 0.14 M, [Catalyst] = 0.007 M, [Coligand] = [4-PPNO] = 0.07 M, [NaClO] = 0.14 M, [Na_2_HPO_4_] = 0.05 M at pH = 11.2 as buffer. ^b^ Determined by GC on a chiral column; *ee* values are referred to the major *cis* epoxide (*ee_cis_*). ^c^ Determined by measuring the optical rotation. ^d^ No coligand added. ^e^ Yield by weight of the isolated product. ^f^ Determined by ^1^H-NMR analysis in the presence of Eu(+)(hfc)_3_.

The observed effect of the coligand (compare entries **1** and **2**) and the absolute configuration of the *cis*-epoxides were in agreement with literature data [14,20,21,22]. 

**Figure 4:**
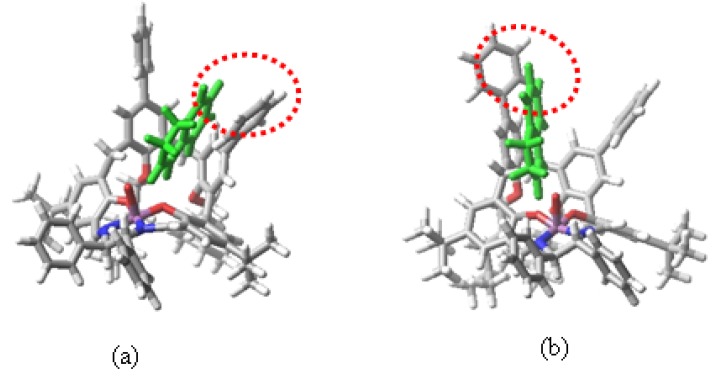
Computer optimized structures concerning the *re* (**a**) and *si* (**b**) face attack of dihydronaphthalene to the **6-Mn** catalyst.

Previously reported studies indicated that the dissymmetry of diimine bridge should favour the approaching of the *si* enantioface of the alkene towards the Mn-oxo site of chiral salen catalysts [14]. The observed *ee* values for the alkenes reported in Table 1 are in line with this finding. However the degree of enantioselectivity displayed is moderate. In order to try to understand the observed behaviour, molecular mechanics calculations were performed on the complexes. Figure 4 shows the attack to the catalyst of both *re* and *si* face of the dihydronaphthalene. Very small differences were found for calculated energies (<5 kJ mol^−1^) suggesting that the attack pathways by the *re* and *si* face are energetically equivalent. Similar results were obtained in all the modelled structures of the examined alkenes and the catalyst. This behaviour might be ascribed to the presence of the biphenyl rings, which can undergo atropoisomerization and then can adapt their spatial position to the approaching guest, favouring therefore π-π interactions with the aromatic framework of the alkene regardless of the alkene exposed face.

## 3. Experimental

### 3.1. General

Melting points were determined on a Kofler hot stage apparatus and are uncorrected. The NMR experiments were carried out at 27 °C on a 500 MHz spectrometer (^1^H at 499.88 MHz, ^13^C-NMR at 125.7 MHz) equipped with pulse field gradient module (Z axis) and a tunable 5mm inverse detection probe. The chemical shifts (ppm) were referenced to TMS as internal standard. Gas chromatographic analyses of the reaction mixtures were carried out on a gas chromatograph equipped with a flame ionization detector and program capability on a DMePeBETACDX (1,2-dihydronaphthalene) (25 m × 0.25 mm ID, 0.25 μm film) against an internal standard (*n*-decane). ESI mass spectra were obtained by employing an ESI-MS spectrometer equipped with an ion trap analyzer. The absolute configuration of (1*R*,2*S*)-1,2-epoxy-1,2,3,4-tetrahydronaphthalene, (3*R*,4*R*)-3,4-epoxy-6-methoxy-2-2-dimethyl chromene, (3*R*,4*R*)-3,4-epoxy-6-cyano-2-2-dimethylchromene and (3*R*,4*R*)-3,4-epoxy-6-nitro-2-2-dimethylchromene were determined by measuring the optical rotation with a polarimeter. Measurements of optical rotation gave [α]_D_^20 ^= +17.5 (*c* = 0.20, CHCl_3_) for 1,2-epoxy-1,2,3,4-tetrahydronaphthalene, [α]_D_^20 ^= +10.8 (*c* = 1.1, CHCl_3_) for 3,4-epoxy-6-methoxy-2-2-dimethylchromene, [α]_D_^20 ^= +34.3 (*c* = 0.94, CHCl_3_) for 3,4-epoxy-6-cyano-2-2-dimethylchromene, [α]_D_^20 ^= +11.1 (*c* =0.1, CHCl_3_) for 3,4-epoxy-6-nitro-2-2-dimethylchromene. Absolute configurations were assigned by comparison of the measured [α]_D_^20^ values with those reported in the literature [14,23]. Commercial reagents were used as received without further purification unless otherwise noted. 6-Cyano-2,2-dimethylchromene, 6-nitro-2,2-dimethylchromene and 6-methoxy-2,2-dimethylchromene were synthesized by the Bergmann and Gericke procedure [24]. Dichloromethane was freshly distilled from calcium hydride before use.

### 3.2. General Procedures for the Epoxidation Reactions

To a stirred solution of alkene (0.35 mmol), catalyst (0.0175 mmol) and 4-phenylpyridine-*N*-oxide (4-PPNO, 0.175 mmol) in CH_2_Cl_2_ (2.5 mL), kept in a round bottom flask and maintained at 25 °C in a thermostatic bath, buffered bleach (0.35 mmol, buffered to pH = 11.2 with 0.05M Na_2_HPO_4_) is added. The course of the reaction was monitored by GC. After 24 h, the phases were separated, and the aqueous layer was extracted with CH_2_Cl_2_. The combined organic phases were dried over Na_2_SO_4_ and concentrated. The crude product was purified by PLC (SiO_2_).

*3-[3-(Hydroxymethyl)-5-phenylsalicyl]-5-phenyl-2-hydroxybenzyl alcohol* (**1**). Compound **1** was prepared according to literature procedure [15]: a mixture of *p*-phenylphenol (30 g, 0.18 mol) and formaldehyde 37% (150 mL, 1.8 mol) was cooled in an ice bath, treated slowly with KOH (20.4 g, 0.36 mol), and then stirred for 7 days at 40 °C. Crystallization from CH_3_OH yielded 30 g (40%) of **1** as a white powder: mp 127–128 °C. ^1^H-NMR (acetone-d_6_) δ ppm: 7.55 ArH (d, 6H, *J* = 7Hz) 7.37 ArH (t, 4H, *J* = 7.4Hz,) 7.32 ArH (s, 2H), 7.24 ArH (t, 2H, *J* = 7.4Hz), 4.90 CH_2_OH (s, 4H), 4.11 CH_2_ (s, 2H), 3.30 OH (br, 2H). ESI-MS : m/z 435 [M Na]^+^.

*3-[2-Isohexyloxy-3-(hydroxymethyl]-5-phenyl-2-isohexyloxybenzyl alcohol* (**2**). A stirred mixture of **1** (5.43 g, 13 mmol), 1-bromo-4-methylpentane (10 g, 61 mmol) and anhydrous K_2_CO_3_ (4.2 g, 30 mmol) in dry MeCN (200 mL) was refluxed for 24 h. After filtration and evaporation of the solvent, the residue was dissolved in CH_2_Cl_2_. The organic layer was washed with water, dried over anhydrous Na_2_SO_4_ and concentrated. Crystallization from CH_2_Cl_2_/n-hexane yielded 5.1 g (67%) of **2** as a white powder: mp 90–91 °C. ^1^H-NMR (CDCl_3_) δ ppm: 7.47 ArH (m, 6H), 7.36 ArH (t, 4H, *J* = 7.5 Hz), 7.28 ArH (m, 4H), 4.81 CH_2_OH (d, 4H, *J* = 6 Hz), 4.17 CH_2_ (s, 2H), 3.86 OCH_2_ (t, 4H, *J* = 6.5 Hz), 2.19 CH_2_OH (t, 2H, *J* = 6 Hz), 1.82 OCH_2_*CH_2_* (m, 4H), 1.59 (CH_3_)_2_C*H* (m, 2H), 1.35 OCH_2_CH_2_*CH_2_*(m, 4H), 0.92 CH_3_ (d, 12H, *J* = 7 Hz).^ 13^C-NMR (CDCl_3_) δ ppm: 155.93, 153.30, 150.94, 142.79, 140.59, 138.07, 133.51, 128.62, 128.07, 127.76, 127.05, 127.02, 126.97, 126.30, 126.22, 124.20, 75.67, 63.68, 35.01, 33.99, 31.55, 29.70, 27.99, 27.92, 22.54; ESI-MS : m/z 603 [MNa]^+^. Anal. Calcd for C_39_H_48_O_4_: C, 80.69; H, 8.28. Found: C, 80.98; H, 8.16.

*2-[3-[3-(5-tert-Butylsalicyl)-5-phenyl-2-isohexyloxybenzyl]-5-phenyl-2-isohexyloxybenzyl]-4-tert-butylphenol* (**3**). A stirred mixture of **2** (5.0 g, 9 mmol), p-tert-butylphenol (13 g, 86 mmol) and *p*-toluenesulfonic acid (370 mg, 0.44 mmol) in benzene (30 mL) was refluxed for 24h. After evaporation of the solvent and remove excess of *p-tert*-butylphenol with sublimate under vacuum at 115 °C the residue was dissolved in CH_2_Cl_2_. The organic layer was washed with water, dried over anhydrous Na_2_SO_4_, concentrated and purified by column chromatography (SiO_2_, eluent *n*-hexane/AcOEt 7:1, v/v) to give (**3**) (5.5 g, 76%) as a white powder: mp 99–100 °C. ^1^H-NMR (CDCl_3_) δ ppm: 7.40–7.12 ArH (m, 18H), 6.78 ArH (d, 2H, *J* = 8.5 Hz) 4.19 CH_2_ (s, 2H), 3.98 OCH_2_ (t, 2H, *J* = 6.5 Hz), 3.95 CH_2_ (s, 4H), 1.90 OCH_2_CH_2_ (m, 4H), 1.56 (CH_3_)_2_CH (m, 2H), 1.35 OCH_2_CH_2_CH_2_ (m, 4H), 1.29 CCH_3_ (s, 18H), 0.92 CH_3_ (d, 12H, *J* = 7 Hz). ^13^C-NMR (CDCl_3_) δ ppm: 152.95, 152.25, 142.97, 140.50, 138.38, 133.62, 133.39, 128.61, 128.10, 127.77, 127.12, 127.02, 126.39, 125.62, 125.11, 116.06, 114.73, 75.88, 34.9, 33.98, 32.25, 31.62, 31.52, 29.98, 27.88, 22.46; ESI-MS: m/z 867 [MNa]^+^. Anal. Calcd for C_59_H_72_O_4_: C, 83.89; H, 8.53. Found: C, 80.87; H, 8.59.

*3-[3-[3-[3-(Formyl)-(5-tert-butylsalicyl)]-5-phenyl-2-isohexyloxybenzyl]-5-phenyl-2-isohexyloxy-benzyl]-5-tert-butyl-salicylaldehyde* (**4**). To a chilled solution of **3** (1.12 g, 1.32 mmol) in dry CH_2_Cl_2_ (50 mL) was added Cl_2_CHOCH_3_ (2.34 mL, 26.4 mmol) under N_2_. A 1M solution of SnCl_4_ in CH_2_Cl_2_ (2.64 mL, 2.64 mmol) was then added and after 20 minutes the reaction was stopped with 1M HCl [14]. The mixture was washed with a saturated aqueous solution of NaHCO_3_ and then with water, and dried over anhydrous Na_2_SO_4_. The solvent was removed under reduced pressure and the resulting oil was purified by column chromatography (SiO_2_, eluent: *n*-hexane/acetone 6:1) to give **4** as a white solid. (0.83 g, 70%). ^1^H-NMR (CDCl_3_) δ ppm: 11.18 ArOH (s, 2H), 9.88 CHO (s, 2H), 7.50–7.18 ArH (m, 18H), 4.22 CH_2_ (s, 2H), 4.12 CH_2_ (s, 4H), 3.84 OCH_2_ (t, 4H, *J* = 6.5 Hz ), 1.90 OCH_2_CH_2_ (m, 4H), 1.59(CH_3_)_2_CH (m, 2H), 1.35 OCH_2_CH_2_CH_2_ (m, 4H), 1.26 CCH_3_ (s, 18H), 0.91 CH_3_ (d, 12H, *J* = 7 Hz). MS-ESI: m/z 923 [MNa]^+^. Anal. Calcd for C_61_H_72_O_6_: C, 81.33; H, 8.0. Found: C, 81.74; H, 8.41.

*Condensation of 3-[3-[3-[3-(formyl)-(5-tert-butylsalicyl)]-5-phenyl-2-isohexyloxybenzyl]-5-phenyl-2-isohexyloxybenzyl]-5-tert-butyl- salicylaldehyde with (1R,2R)-diphenylethylendiamine (5)*. Solutions of **4** (0.27 g 0.3 mmol) in EtOH (27 mL) and (*1R*,2*R*)-1,2-diphenylethylendiamine (0.064 g, 0.3 mmol), in EtOH (14 mL), were dropped separately but synchronously from two dropping funnels into boiling abs EtOH (200 mL) under rapid stirring. The reaction mixture was refluxed for an additional 24 h, and cooled. After removal of the solvent, the crude product was column chromatographed (SiO_2_, eluent *n*-hexane/acetone 6:1, v/v) to afford **5** (0.13 g, 40%). ^1^H-NMR (CDCl_3_) δ ppm: 13.36 ArOH (s, 2H), 8.42 CH=N (s, 2H), 7.37–7.00 ArH (m, 26H), 4.73 CH-N (s, 2H), 4.26 CH_2_ (d, 2H, *J* =15 Hz), 4.23 (s, 2H), 4.04 CH_2_ (d, 4H *J* =15 Hz), 3.83 OCH_2_ (t, 4H, *J* = 6.5 Hz), 1.79 OCH_2_CH_2_ (m, 4H), 1.50(CH_3_)_2_CH (m, 2H), 1.30 OCH_2_CH_2_CH_2_ (m, 4H), 1.1 CCH_3_ (s, 18H), 0.8 CH_3_ (d, 12H, *J* = 7 Hz).^ 13^C-NMR (CDCl_3_) δ ppm: 166.76, 156.722, 155.71, 141.14, 140.94, 139.56, 136.54, 134.30, 133.85, 131.09, 128.84, 128.49, 128.25, 128.00, 127.71, 127.51, 127.39, 126.93, 126.58, 126.44, 117.55, 35.17, 33.80, 31.39, 31.31, 30.94, 29.31, 28.35, 27.96, 22.55; ESI-MS: m/z 1077 [MH]^+^. Anal. Calcd for C_75_H_84_N_2_O_4_: C, 83.64; H, 7.81; N, 2.60. Found: C, 83.31; H, 7.86; N, 2.62.

*Uranyl complex* (**6-UO_2_**). (AcO)_2_UO_2_•2H_2_O (10 mg, 0.025 mmol) was added as a solid to a stirred solution of the ligand **5** ( 20 mg, 0.02 mmol) in MeOH (10 mL). The mixture was allowed to stir overnight at room temperature and was monitored by TLC (eluent: 20% acetone in *n*-hexane). The solvent was removed on a rotary evaporator under vacuum and the residue was dissolved in CH_2_Cl_2_, filtered and concentrated to produce the pertinent uranyl complex in a nearly quantitative yield. ^1^H-NMR (acetone d_6_) δ ppm: 9.41 CH=N (br, 2H), 7.79–7.08 ArH (m, 26H), 6.23 CH-N (s, 2H), 4.52 CH_2_ (s, 2H,), 4.28 CH_2_ (s, 4H), 3.96 OCH_2_ (bt, 4H), 1.82 OCH_2_CH_2_ (s, 4H), 1.65 (CH_3_)_2_CH (m, 2H), 1.35 OCH_2_CH_2_CH_2_ (m, 4H), 1.26 CCH_3_ (s, 18H), 0.91 CH_3_ (d, 12H, *J* = 7 Hz). ESI-MS: m/z 1345 [MH]^+^. Anal. Calcd for C_75_H_82_N_2_O_6_U: C, 66.96; H, 6.10; N, 2.08. Found: C, 66.73; H, 6.13; N, 2.10.

*[Mn(III)(L)] complex* (**6-Mn**). To a solution of the ligand 5 (65 mg, 0.06 mmol) in CH_2_Cl_2_ (8 mL) was added a solution of Mn(OAc)_3_•2H_2_O (16 mg, 0.064 mmol) in EtOH (8 mL). The dark solution was allowed to stir overnight at room temperature and was monitored by TLC (eluent: 20% acetone in *n*-hexane). The solvent was removed under vacuum and the residue was dissolved in CH_2_Cl_2_, filtered and concentrated to produce the Mn(III) catalyst in a nearly quantitative yield. ESI-MS: m/z 1129 [M]^+^. Anal. Calcd for C_77_H_85_MnN_2_O_6_: C, 77.78; H, 7.15; N, 2.36. Found: C, 77.68; H, 7.20; N, 2.40.4.

## 4. Conclusions

We have synthesized a new macrocyclic chiral salen ligand and its UO_2_ and Mn(III) complexes. NMR studies of the (salen) UO_2_ complex are in agreement with MM calculated structures which indicate that the shallow conformation of the receptor cavity and, probably, the mobility of biphenyl rings are responsible of the observed moderate enantioselectivity. At any rate, the catalyst synthesized in this work represents the first example of salen derivatives combined with biphenyl units and the work is in progress to avoid the atropoisomerization of biphenyl units, in order to control more efficiently molecular recognition.
